# Cases of Purpura Hæmorrhagica, with Observations on the Pathology and Treatment of the Disease

**Published:** 1878-06

**Authors:** N. S. Davis

**Affiliations:** Chicago, Ill.


### CASES OF PURPURA HÆMORRHAGICA, WITH OBSERVATIONS ON THE PATHOLOGY AND TREATMENT OF THE DISEASE.

By N. S. Davis, M. D., Chicago, Ill.

(*Read to the Chicago Medical Society, April, 1878.*)

Cases of simple purpura, or purple spots accompanied by moderate cellular inflammation and slight general febrile disturbance, are not very infrequent; neither is it rare to find cases presenting petechial and ecchymosed spots upon the cutaneous surface connected with various pathological conditions of the blood and tissues, of a temporary character. But cases of true purpura hæmorrhagica arising from a more or less permanent hæmorrhagic diathesis, or what is called in the seventeenth volume of Ziemssen's Cyclopædia, hæmaphilia, are fortunately, rarely met with. This is shown by the fact that Grandidier, who has given diligent attention to the literature of this subject, found only 631 reported cases prior to 1872, and Immermann, in his recent article has added only 19 more, making a total of 650 cases. During the twenty-nine years that I have practiced in this city, I have notes of only five well marked cases coming directly under my own observation. The first of these was a boy aged twelve years, who had commenced the trade of stone cutter, but of whose parentage or family history I obtained no record. At the time he came under my observation, he was suffering from extensive hæmorrhage into the skin and subcutaneous areolar tissues. He presented not less than four blood-tumors, hæmatoma, varying in size from one to six inches in diameter. The largest was on the posterior part of the chest near the lower angle of the scapula. Besides these tumors there were large numbers of ecchymosed and petechial spots, both on the body and limbs. He was tall, spare in flesh and moderately anæmic in appearance, but the action of the heart was increased both in force and frequency. He complained of great weakness, but had no external hæmorrhage. He began to exhibit the hæmorrhagic tendency when he was between five and six years of age. The slightest cut or injury would bleed in the most persistent manner; and any severe muscular exercise was sure to induce hæmorrhagic extravasations into the subcutaneous tissues. By rest, mild diet, and the use of astringents and tonics, he recovered from the attack for which he first came under my observation; but other attacks less severe occurred three or four times during the next two years, after which I lost all further knowledge of his progress.

The *second* case was a boy aged five years, brought to me from Peoria. He had had frequent and persistent attacks of both epistaxis and interstitial hæmorrhages for two years or more, and was at the time of coming under my care exceedingly feeble. He had an anæmic appearance; rheumatic pains in his limbs with some serous effusion into the synovial membranes of the knee and ankle joints; numerous petechial and ecchymosed spots in the skin; a quick and irritable pulse with increased cardiac impulse, but no valvular murmurs.

He was kept on a mild diet, chiefly of farinaceous articles and milk; and the use of suitable doses of digitalis, ergot, and tincture of the chloride of iron several months, during which his health and strength much improved, and his hæmorrhages were much less frequent. His subsequent progress I have never learned.

The *third* case was that of a boy aged only two and a half years, living on Halsted, north of Division street, in a poor family of Irish parentage. I was first called to see him eight years since. At that time blood was oozing from the gums around the two back teeth on one side of the mouth, from the edge of the tongue, and from the nose. It had been in progress more than a week and he was much exhausted. His knees and ankles were some swollen and his face appeared puffy and bloated, with numerous petechial spots on the skin; pulse frequent and weak, but the heart's action increased. His mother stated that he began to exhibit a disposition to persistent bleeding on the slightest accident before he was a year old, and for several months past had bled so often from the nostrils that she had little hopes of his recovery. For the arrest of the existing hemorrhage I directed a solution of persulphate of iron in water, in such doses that he would get half a grain every three hours, and tincture of digitalis and fluid extract of ergot, each two minims, half way between the doses of the iron. He was kept at rest and fed on bread, rice and milk. In about two days the external bleeding ceased, and soon after the petechial spots began to fade. The tincture of chloride of iron was then given instead of the persulphate, and a minute dose of strychnia added to each dose of the iron, giving it only after each meal-time. The digitalis and ergot were continued before each meal and at bed-time. He continued to improve until in about four weeks all appearance of hæmorrhagic spots had disappeared and his general health was good. The iron and strychnia were then discontinued, but the digitalis and ergot were continued with only intervals of three or four days at a time for more than a year. During the first half of the year he had three or four moderate turns of epistaxis, and during the last six months no spontaneous hæmorrhages. Every bruise or slight scratch, however, still showed the existence of the diathesis. The mother was now instructed to give him only one dose of the digitalis and ergot at bed time each night; to guard him as fully as possible against all traumatic injuries, and to give suitable doses of the medicine four times a day whenever any appearance of hæmorrhage, either spontaneous or traumatic, should occur. The mother followed the directions faithfully for three years, during which he grew so well and had so little trouble that further use of medicine was omitted. He continued to do well until about one year since, when the mother brought him to my office with epistaxis which had persisted moderately three days; two or three blood tumors on his body and thighs, with several petechial spots in the skin. I directed a return to the use of the same remedies as before, in doses suited to his increased age, and the bleeding was again arrested, and I have heard nothing from him since.

The *fourth* case was a boy on Lytle street, in the West Division of the city. He came under my observation when he was about four years of age. The hæmorrhagic tendency had been observed from his infancy, and was so strongly marked that his bleedings were both external and interstitial, provoked by the most trifling causes, and persisting several times until death seemed almost inevitable. I recommended the same general management hygienic and medical as in case three, just detailed. He has been somewhat under my observation for four or five years. The treatment had the influence to greatly improve his condition, lengthening the intervals between his hæmorrhagic attacks, and rendering them less persistent, but the last time I saw or heard from him, which was about one year since, he was suffering from an extensive hæmorrhagic extravasation into the areolar tissue of one leg and thigh, with severe rheumatic pains.

The fifth and last case is a young woman aged about 18 years, who first came under my observation last winter, in the early part of February. She had been bleeding from the nostrils so freely for several days that her face was very pale, and the physician in attendance had plugged both posterior and anterior nares, and was giving her half-drachm doses of fluid extract of ergot every two hours. Her pulse was feeble, extremities cold, the skin on the upper and lower extremities exhibiting numerous small petechial spots with several much larger ones on the trunk of the body, and a feeling of soreness or muscular hyperæsthesia throughout the system. Her menstrual functions had been regular as to time and only slightly more free than the average of healthy women. There was nothing in her mode of living or in the appearance of her gums to indicate a scorbutic tendency.

As the plugging of the nostrils had stopped the direct flow of blood, she was advised to continue the use of the ergot in doses of fifteen minims of the fluid extract in combination with the same quantity of the tincture of digitalis, every four hours. Entire rest and mild diet were enjoined, and she soon began to improve. After a week had passed with no return of the bleeding and the petechial spots had begun to fade, the ergot and digitalis were restricted to a dose three times a day and ten drops of dialyzed iron and one-thirtieth of a grain of strychnia were given after each meal time. In about three weeks the hæmorrhagic spots had all disappeared and the patient appeared quite well. She continued so until the second week in March, when she again began to be troubled with persistent epistaxis. It was controlled however, before she became greatly reduced by the internal use of ergot and digitalis with rest. This patient had been subject to hæmorrhagic attacks once in two or three months for the last three years.

The character of the bleeding in cases of true hæmaphilia is somewhat peculiar. Instead of coming from one or more distinct vessels, it oozes from the whole bleeding surface, whether it be that of a wound or a membrane.

The place from which the bleeding comes may be either internal, interstitial, or external from free surfaces. Of the latter the relative frequency of the bleeding from different parts is shown by the following statistics given by Immermann: In 308 cases, 152 bled from the nose; 38 from the gums; 35 from the intestines; 17 from the lungs; 16 from the urinary organs; 14 from the stomach; 10 from the female genital organs; 6 from the tongue; 5 from the ears; 4 from the fingers; 4 from the scalp; 3 from the caruncula lacrymalis; 2 from ulcers of the skin; 1 from the eyelids, and 1 from the umbilicus.

It will be seen that epistaxis greatly predominates in frequency; yet the bleedings may occur from almost every part of the body.

In regard to the causes contributing to the formation of the hæmorrhagic diathesis but little is known. That the cases are in large part congenital is evident from the early age at which the disease first manifests itself. Thus of 95 cases collected by Grandidier, 58 began before the completion of one year of age, and 23 began between the ages of one and six years; to which may be added 4 of the 5 related in this paper. In regard to hereditary influence, it appears that the 650 cases compiled by Grandidier and Immermann belonged to 219 families, being in the ratio of nearly three in each family. The influence of sex is still more strikingly illustrated by the same 650 cases, of whom 602 were males and only 48 females; and 4 of the 5 cases related by myself were males.

*Pathology of the Disease.*—By some, it has been regarded as a morbid condition of the blood, consisting *in deficiency* of hæmatine or red corpuscles, fibrin and plastic constituents. But recent careful analyses have shown that these ingredients, especially the iron and fibrin are in excess instead of being deficient in quantity; and consequently investigations in this direction afford no explanation of the morbid phenomena. Careful post mortem examinations have shown in some cases, cardiac hypertrophy with or without enlargement of the aorta; in others the blood vessels were represented as lying more superficial and with thinner coats, but with less capacity of the arterial termination in the capillaries. It is acknowledged that these anatomical changes or defects are not present in all cases and that the last named is very difficult to determine. Yet on this vague idea of the want of capacity in the termini of the vessels, Immerman founds his explanation of the phenomena of the disease. He claims that there is in hæmaphilia generally an excess of blood or plethora of the vessels and a remarkable rapidity of hæmatosis or reproduction of blood after hæmorrhages; and this in conjunction with the defective capacity of the peripheral extremities of the vessels leads to undue lateral pressure upon their walls and the consequent forcible exudation or extravasation of blood.

If this theory was correct, the periods of hæmorrhage ought always to cease, as soon as sufficient blood had been lost to relieve the vascular fullness and consequently the lateral pressure, which is not in accordance with clinical observations.

It has appeared to me, that the pathological defects or conditions in these cases did not consist in a disproportion between the *quantity* of the blood and the *capacity* of the peripheral extremities of the vessels, but in, either the defective development of the muscular fibers in the middle coat of the smaller arteries, or the defective influence of the vaso-motor nerves on such fibers, or both combined. Another element that I think enters into most of these cases is a defect in the property or affinity belonging to the plastic elements of the blood and the tissues.

We all know that when a part is irritated, among the earliest changes noticable under the microscope are the rapid accumulation and adhesion of the white corpuscles to the walls of the vessels, which together with the constant processes of nutrition and disintegration involving definite atomic changes, prove the existence of a special affinity as a property of living plastic elements.

It is not the mere retraction of a severed vessel and the formation of a clot that arrests hæmorrhage in healthy subjects. On the contrary, I am satisfied that injury to a vessel in a previously healthy state induces a reflex influence through the vaso-motor nerves, causing tonic contraction of the muscular fibers of the vessel or vessels implicated, and this, with the rapid accumulation and adhesion of corpuscles and plastic constituents to the walls of the vessels, has far more to do with the permanent suppression of hæmorrhage than the mere retraction of the vessel and formation of the clot. If I am right in this view, it is easy to perceive that a defect in the development of the muscular fibres of the vessels with an impairment or paralysis of the vaso-motor nervous influence, whether congenital or acquired, would constitute a condition highly favorable for the occurrence of frequent and persistent hæmorrhages.

*Treatment.*—As would be inferred from the cases reported in the first part of this paper, I place more reliance on the use of digitalis and ergot, internally, aided by a solution of persulphate of iron applied to the bleeding surfaces, than on any other remedies. During a period of actual bleeding, the remedies should be given in pretty full and frequent doses, aided by entire rest and mild diet. After the bleeding ceases I continue the same remedies, in smaller doses, three times a day, from three to six months, interrupting them occasionally for three or four days at a time. Careful attention should also be given to keeping the digestive and excretory organs in good order, and there should not be too much haste in promoting the re-formation of blood by resorting to rich food and preparations of iron. Reliable analyses have shown that both iron and fibrine are generally present in the blood of these patients in quantity above the normal proportion, and the heart is unduly excitable. This would indicate a diet of farinaceous articles, milk and vegetables, with only a sparing use of meat, and such nervous excitants as tea and coffee; and my own experience has fully corroborated this view. I have also found it advantageous to limit the patient in the use of water or other drinks habitually, because free indulgence in this respect rapidly increases the mere bulk of the blood without adding to its plastic elements.

